# Review of "Therapy, Sensory Integration and the Autistic Child" by DS. Berger

**DOI:** 10.1186/1475-925X-4-9

**Published:** 2005-02-10

**Authors:** Max E Valentinuzzi

**Affiliations:** 1 Department of Bioengineering, Instituto Superior de Investigaciones Biológicas (INSIBIO), Consejo Nacional de Investigaciones Científicas y Técnicas (CONICET), Argentina

Innumerable animal species produce noises of different kind and many of them sound musical to us – human beings – eliciting a variety of emotional feelings, as the frogs' chants in a rainy night, or the wild birds' songs in a sunny morning perhaps mixed with our finch's joyful trills from its small cage, or the deep thrilling whales' hissing who very few have had the chance to listen (as I once did in Colombia). Most likely, they all are just communication ways, perhaps to let the other guy know that the male is there, waiting for the female to come and meet it, because usually only males sing. Is all that really music? I do not think so, for music is unique to man and woman and only we, human beings, get together either in small or large groups just to listen to music or to make music. Music conveys a message, which only the human being can decipher in his/her own fashion; as in animals, it is no doubt a communication means. The isolated man or woman, the Robinson Crusoe, is essentially non-existent. Music is a natural human need, almost at the level of the basic biological ones (self and species preservation).

The current literature on music and the human being is enormous, from artistic to technical to psychological to physiological aspects, or what have you. Music is so much embedded in us that never many studies are or will be enough. This little book, little in size but not in scope, authored by Dorita S. Berger (an accomplished concert pianist and certified music therapist, with superb academic background and solid experience), seems to cover a paucity because it falls within the reach of those who, without the requisite of being specialists are not fully outsiders; it also serves as an introduction to those who may like to proceed further in any of the several paths that converge and diverge to and from this fascinating area of human adaptation. The book consists of a foreword, a preface, 11 chapters and 2 appendices. Its foreword is unique and deep in content, indeed. Written by Donna Williams, herself a diagnosed autistic, captivating and exemplary woman, author of several publications (among them a book entitled *Autism: An Inside-Out Approach*, 1996, Jessica Kingsley Publishers), its sentences must be read over and over until one really apprehends its profound meaning, as her initial paragraph, where she states: "Mind and consciousness are like a gradual sunrise that takes time to come up. In the meantime, we are not stagnant, waiting for life to happen." She refers to "the music of life, felt with our bodies, long before we identify mind with self ... ... some of us stay here longer than others." And a little further down, "music in its broader sense is everywhere, and it is our first language ... it has the most important of all places in the lives of those who find the realm of the mind a place of rusty cogs and heavy effort." Donna declares that music was an exceptionally important part of her life and almost naïvely and charmingly confesses: "I could not understand three sentences in a row until I was nine". These deep thoughts and experiences remind of James Joyce's *agenbite of inwit*, or the wound of in-looking, the hurt of self-analysis, the painful knowing of oneself, introduced in his famous, conflicting and monumental *Ulysses*. Man at large is the prey of inner suffering.

The preface is already in the hands of the author, where the book is introduced along with some general concepts, such as: "Entering the brain through the auditory system is a key to obtaining automatic brain attendance and response" and "physiologic changes resulting from music intervention (or stimulation) can be a major factor in bringing about a feeling of well-being". Dorita aims the text at music therapists, parents, teachers and health clinicians. However, in my opinion and as anticipated above, its audience should not be restricted to such relatively small group but rather can be much broader, as there are, for example, students or even researchers developing specific projects in the area. Already ending the preface, the author underlines quite sensibly that the book is not a recipe since music therapy is an approach to solving a physiologic or psychological problem.

Chapter 1 (Introduction: Who Defines "Appropriate"?, pp17–25) is a dareful good beginning that encourages the reader to proceed. For the human animal, the expectation is to "follow the rules" of the pack and behave accordingly, and Dorita very wisely asks who defines "appropriateness" and for whose benefit the rules are designed. What is normal and typical? Are the two words interchangeable? What determines "right" and "wrong" responses? Are these not "labels" somehow imposed by society? The concepts of "right" and "wrong" are basically determined by socio-cultural standards rather than scientific givens. All this set of questions go far beyond the intended objective of the book and scrape deep in the human self, both as an individual and as a member of society. Allow me to bring about the painful, senseless, dire and cruel Irak War (April 2003): Is the Bush administration not imposing its own pack rules, denying rights as illegal for other countries (other societies or packs) while retaining those very same rights as legal for itself? Is it bad behavior for the others to have weapons while it is good behavior for the USA to have them? No doubt, Dorita touches, perhaps without realizing, a huge and essentially unsolvable problem that only true generosity and unabashed love, mostly and so far unsuccessfully called for by the core of our unfulfilled religions, might one distant and dreamt of day bring forth. Yes, excellent and well-posed question: Who defines "appropriate"?

In Chapter 2 (Aspects of Autism, pp26–34) the author says that autism is a *neurologically atypical *manner of function, with genetic and sensory-motor implications; what appears in childhood, remains throughout life. She introduces the concept of a different world the child is immersed in and, as such, cannot possibly get connected to this "real" one, with other channels and incomprehensible rules. His/her desire for sameness is broken at worst or disturbed at best. Another good point: By and large, most of the people avoid anything bringing change, anxiety, confusion, insecurity and the like. Sameness, conservation of what we know, appears as safer. A trip to a foreign country or place carries with its sole organization an element of tension and we often think "oh, we had better call it all off". And children are particularly susceptible and sensitive to these changes (such as a new school or neighborhood).

Chapters 3 (Aspects of Sensory Integration, pp35–48), 4 (Functional Adaptation Defined, pp 49–60), 5 (Understanding Basic Sensory Systems, pp 61–77) and 6 (Are You Listening? Part One: About Hearing and Listening, pp78–90) deal mainly with physiological aspects, perhaps a little in excess because it tends to overload the reader removing him/her from the central subject, which is music, as stimulus or intervention, akin in a sense to electrical or mechanical or chemical or thermic or magnetic stimulation. However, it is fine and useful. As an interested reader and somewhat also as an actor within the discipline, the comment may be taken simply as an expression of desire. One paragraph called my attention almost at the end of chapter 3: "Music does not require semantic interpretation. It simply provides an environment – a sound blanket – wrapping itself around the body and providing a sense of safety and security." Good attractive concept, but I would emphasize what I say previously: Music carries a message, it may not be semantic or absolute, but the listener somehow decodes it to suit his/her own cenesthetic state.

The basic driving forces of man and woman are clearly stated in Chapter 4, two are purely biological (as anticipated above); other two pertain only to the human being, *self-determination *or *freedom of choice *and *spiritual fulfillment*. Let me add that one essential tool for their satisfaction is *communication*, which is a need, and music appears as a phenomenal way to accomplish it. The messages left by Johann Sebastian Bach, Wolfgang Amadeus Mozart or Ludwig van Beethoven are there and will be there forever, hence demonstrating unparalleled self-determination and spiritual fulfillment, even after death. Finally, accommodation, as discussed here, is a good classical physiology concept that fits very well this new frame of music and human adaptation. An appealing idea advanced in Chapter 5 refers to a possible release of chemical neurotransmitters (such as dopamine, endorphines, encephalines or others) elicited by music stimulation. The whole thing may mark a difficult but attractive research avenue.

Chapter 6 is in my modest opinion and with due respects the weakest of the book: auditory scanning is definitely obscure, the section titled "sound coding tango" (what does the word "tango" do here?) does not show a relationship with the text that follows and the remaining sections are at best confusing. The last paragraph in page 89 sounds very appealing, at least to me, and I tend to agree with it ... but it lacks so far solid scientific support.

In Chapter 7 (Are You Listening? Part Two: Dimensional Hearing and Erroneous Assumptions, pp 91–111) Dorita starts with the case of a four year old boy, Jason. No musical instrument really moved him, until one day he spotted a gong. It captured his love and reacted as fully unfazed by its intense volume. Quite an amazing response considering that most people (the pack) get annoyed by a gong sound, especially if it is played within a small room. Thereafter, she briefly discusses some common erroneous assumptions, as for example, "music is always fun" and "the content of a recognizable tune is being perceived by another in the same format in which it is being presented by the therapist". The latter agrees with the previous contention: each listener to suit his/her cenesthetic needs decodes the musical message. Such assumptions deserve to be taken into consideration in other environments, too. Example: A person complains that his neighbor plays piano every day after midnight and, thus, disturbs his sleep. The player argues back saying that he plays beautiful classic music loved by everybody. Who is right? The author brings also forward an interesting and novel possibility: Is there such a thing as *auditory dyslexia*? There seems to be no term for a phenomenon in which sounds are retrieved in incorrect order (as in reading or writing dyslexia). Researchers may have in this another subject to investigate. After finishing this chapter and reviewing the variety of concepts and subjects touched in it, I could not but wondering that the human being enjoys with the eyes the *landscape *he or she moves about, simultaneously the complex auditory tract perceives the *soundscape *that surely accompanies the former, probably also seasoned by an *odorscape*, *tastescape *and even a *touchscape*; but everything is slowly evolving in the *timescape*, as a multimensional function of time. The whole configures the complex scenery we live in creating what Donna calls the music of life.

Chapter 8 (Elements of Music for Sensory Adaptation, pp 112–129) goes, with some tardiness in the development of the book (at least to my particular taste), into music itself, and I would like to use the term *music stimulation*, characterized by six properties, as Dorita explains: *rhythm*, *melody*, *harmony*, *dynamics*, *timbre*, and *form*. This is not the place to give details about these elements for the author clearly fathoms in them solidly and without a single doubt. My only comment, and just as a curiosity recalling my days as cardiovascular physiologist, refers to one teacher actually using pulsed hand-clapping (a pacemaker) to call her entire class of children to order, to subdue chaotic impulses (page 114). A fibrillating heart has its fibers in a dynamic state of dyssynchrony (as the children shouting in the classroom). No effective contraction to eject blood takes place. One or may be two electric shocks usually stop the disorder and the myocardial mass resumes its rhythmic synchronic activity. This is called a defibrillating maneuver. Are the similarities of nature not nice? As Albert Einstein used to say, the most incomprehensible thing of the world is that the world is comprehensible.

Chapter 9 (Music Therapy in the Realm of Sensory Integration, pp 130–151) is more practical as it discusses the different types of musical instruments from the viewpoint of their suitability to specific therapeutic objectives. In Chapter 10 (Formulating Music Therapy Treatment for Sensory Adaptation Goals, pp 152–165), instead, several aspects are discussed, some of them somewhat superficially and not meeting the expectations developed by the subtitles, as for example "adaptive responses to environment of auditory and visual stimuli" or "auditory integration and differentiation". The last Chapter 11 (Conclusions, pp 166–175) is some kind of summary. Let me quote a wise paragraph, a *caveat emptor *or let the buyer beware: "Music therapy does not lend itself readily to rigorous scientific explanation. Findings are anecdotal at best, and based on observations, insights and informed assumptions. Music therapy does not replace other interventions, nor does it need to copy the goals of other therapies in order to be effective". This is why the field is fully open for serious research.

I think the book meets the intent of its author (page 173): [It] "is an introduction to physiologic information and perspectives that can help the music therapist assess more astutely the behaviors and presenting problems of an atypically functioning patient."

Now, and to finish this review by being a little prickly but honestly (and perhaps naïvely) constructive, let me point out a few minor aspects that might improve another edition of the book: The title does not properly reflect the content of the book. It should be named **The Autistic Child, Sensory Integration and Music Therapy**, for this is really the order followed in it. The physiology section could be reduced. There are also some unnecessary repetitions. The bibliography is given in three groups: specific references to each chapter, recommended references, and a general list at the end of the book. It is not practical. I would rather have a single list at the end properly referred to in the course of the text. Appendix A is the reproduction of an article by Schneck and Berger. It adds something to the book but not to its core material. Thus, it might be deleted in the future. Appendix B is a collection of four case histories. By far is too long containing unnecessary information. It should be greatly reduced and limited to the pertinent information. The music portion could be enhanced.

Having said more than enough, I recommend the book and congratulate the author for her dedication, effort and beautiful activity by combining art with benefit to the many time forgotten children.

